# 

**Published:** 1895-01-26

**Authors:** 


CONTENTS.

**Professional Infamy: The Medical Council and the Obstetrical Society** - 287
**Heredity in Consumption** - 288
**Therapeutics and the Lesser Circulation.**—By SIR BENJAMIN WARD RICHARDSON, M.D., F.R.S. - 289
**Some Imperfections of Medical Education** - 290
**Notes and News** - 292
**Medical Progress and Hospital Clinics:**
Skin Grafting and Allied Procedures - 293
PROGRESS IN MEDICINE.—Diseases of Children - 294
PROGRESS IN SURGERY.—Rectal Surgery - 296
NEW DRUGS AND PREPARATIONS - 297
**Annotations.**—Marriage or not Marriage for Women?—A Song or a Sermon?—Is London Healthy? - 291
**Medical News from India** - 298
**Editor's Letter Box.**—"General Hospitals and Infectious Cases." - 298
**The Institutional Workshop:**
**The Financial Strength of Our Voluntary Charities** - 299
PRACTICAL DEPARTMENTS.—Rain Baths - 300
HOSPITAL FESTIVALS AND MEETINGS - 301
**Scraps and Gleanings** - 302
**The Book World of Medicine and Science** - 303
**The Student of Medicine** - 303

### EXTRA SUPPLEMENT.—THE NURSING MIRROR.

**News from the Nursing World.**—The London Hospital.—Matrons under the Poor Law.—A Novel Party—Queen's Nurses—Wanted: A Night Nurse—Grateful Patients.—Darlington.—Nurse or Washerwoman?—A Cottage Hospital—Private Nursing—Crediton Sick Nurse—Dutch Progress—Short Items - cxxv

**Elementary Anatomy and Surgery for Nurses.** By W. MCADAM ECCLES, M.B., B.S., F.R.S. III. The Osseous System (continued) - cxxvii

**Nursing in Japan** - cxxviii

**Asylum News and Nursing** - cxxix

**Dress and Uniforms** - cxxx

**Metropolitan Asylums Board—South Eastern Hospital, New Cross** - cxxxi

**The Government Civil Hospital, Hong Kong** - cxxxi

**An English View of American Nursing** - cxxxii

**Where to Go** - [illegible]

**French Schools for Trained Nurses: Their Origin and Organisation** - cxxxiii

**Wants and Workers** - cxxxiii

**Everybody's Opinion** - cxxxiv

**For Reading to the Sick** - cxxxiv

### FERRIS and COMPANY'S

### PERPETUAL MEDICAL VISITING LIST AND DAY BOOK.

Arranged in Monthly or Weekly Sections for any number of Patients and for any year.

**Size (closed)** ... ... ... ... ... **6⅝ in. by 4⅛ in.**

*"A" Pattern, Monthly Sections*

*"B" Pattern, Weekly Sections* } *When ordering, please state which is required.*

**Reduced Price, complete with two Refills, 5s. 6d. post free. Refill Books, 6d. each. (Packet of six Refill Books post free for 3s.)**

**N.B.**—We shall be happy to forward one of the Visiting Lists to any Medical Practitioner for inspection, on the condition that, if not approved, it is returned within three days.

"*December 30th*, 1893.
"DEAR SIRS,
"I beg to enclose you P.O. in payment for the Visiting List you sent me.
"I consider the List an extremely neat and convenient one. I have shown it to Dr. M——— of this town, and I believe he intends ordering a similar one from you.
"I am, yours truly,
"———, M.A., M.D., M.B., B.Ch."

"DEAR SIRS, "*May 10th*, 1894.
"Please send me half-dozen refills of your Perpetual Medical Visiting List. It is the best Visiting List I have met with. For portability and compactness it is unsurpassed.
"Yours truly, "———, M.B.Ed., C.M."

"DEAR SIRS, "*May 13th*, 1894.
"Please send me half-dozen refills for Visiting List. It gives me great satisfaction. I find it much more convenient than the form I was using previously.
"Yours truly,
"———, M.B.Aber., C.M."

*From the "British Medical Journal," October 13th*, 1894.

"These visiting lists or day-books are made in two forms, one for the list to be made out once a week, the other once a month. Both are of the 'perpetual' type, that is, they will do for any year, and as many pages as are required may be used for any month or week. They are bound up in thin volumes, easily carried in the pocket, so that one is not burdened on one's rounds with the whole year's accounts. They are clearly, one might even say daintily, ruled, and for any practitioner who eschews the old bad plan of making separate charges for each draught and mixture he dispenses, ample space is afforded for any such code of signs as he may invent to indicate his visits, medicines, and consultations."

**Publishers: FERRIS and COMPANY, UNION STREET, BRISTOL.**

CARRYING 40 56lb. WEIGHTS

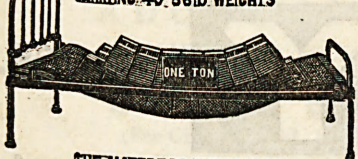

THE MATTRESS RETURNS TO ITS ORIGINAL POSITION

WHEN THE WEIGHTS ARE REMOVED

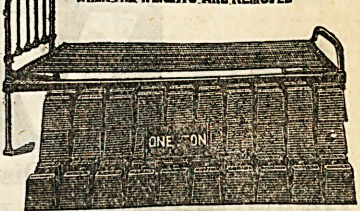

HOSPITAL G WITH LIFTER

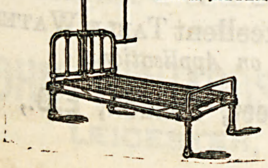

### WOODS' PATENT
### GALVANISED SPRING STEEL WIRE MATTRESSES

(WOOL AND HAIR MATTRESSES).
**Five Years' Guarantee.**

Exhibited, carrying ONE TON, at the Liverpool and Manchester Exhibitions; also at the Meetings of the Medical Association at Dublin, Glasgow, Leeds, and other Exhibitions.

*For Domestic and Hospital Use apply for Catalogues to*

**THE LONGFORD WIRE IRON AND STEEL CO., Ltd., Warrington,**

Which will be sent free on application.

FOR PARALYTIC

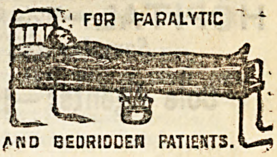

AND BEDRIDDEN PATIENTS.

